# *QuickStats:* Percentage* of Adults Aged ≥18 Years Who Had Lower Back Pain in the Past 3 Months,^†^ by Sex and Age Group — National Health Interview Survey,^§^ United States, 2018

**DOI:** 10.15585/mmwr.mm685152a5

**Published:** 2020-01-03

**Figure:**
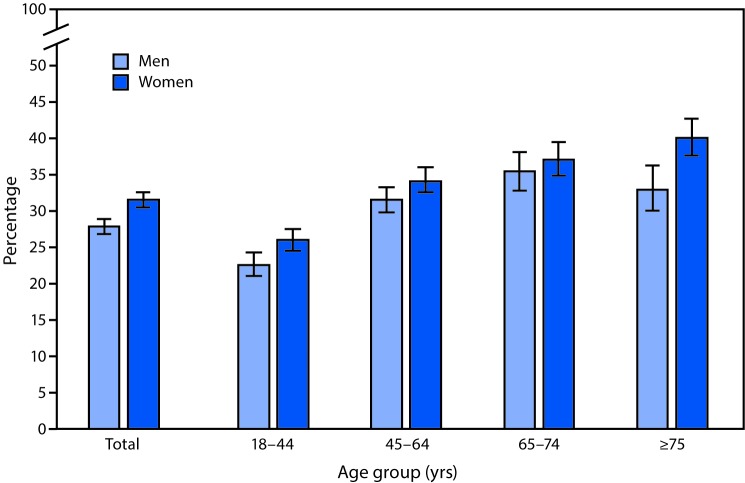
In 2018, 28.0% of men and 31.6% of women aged ≥18 years had lower back pain in the past 3 months. The percentage of women who had lower back pain increased as age increased. Among men, the percentage increased with age through age 74 years and then decreased. Women in the age groups 18–44, 45–64, and ≥75 years were more likely to have lower back pain in the past 3 months than were men in the same age groups, but percentages were similar between men and women in the age group 65–74 years.

**Source:** National Health Interview Survey, 2018. https://www.cdc.gov/nchs/nhis/index.htm.

